# Black Soldier Fly Larva Oil in Diets with Roughage to Concentrate Ratios on Fermentation Characteristics, Degradability, and Methane Generation

**DOI:** 10.3390/ani13152416

**Published:** 2023-07-26

**Authors:** Rittikeard Prachumchai, Anusorn Cherdthong

**Affiliations:** Tropical Feed Resources Research and Development Center (TROFREC), Department of Animal Science, Faculty of Agriculture, Khon Kaen University, Khon Kaen 40002, Thailand; rittikeard1994@gmail.com

**Keywords:** black soldier fly larva, fermentation, volatile fatty acid, protozoa, methane

## Abstract

**Simple Summary:**

Animal husbandry costs are being driven up by a scarcity of high-quality, expensive animal feed. Researchers are investigating alternative feed resources derived from black soldier fly larva, including the utilization of oil from black fly larvae as a byproduct of the industry. A study using in vitro gas production techniques investigated the influence of black soldier fly larva oils and the proportion of roughage-to-concentrate ratios on in vitro fermentation. Results showed that adding 4% black soldier fly larva oils (BSFO) in concentration at different roughage (R)-to-concentrate (C) ratios increased propionate levels, decreased methane (CH_4_) emission, and preserved dry matter (DM) degradability.

**Abstract:**

Currently, the scarcity of high-quality, expensive animal feed is a primary factor driving up the cost of animal husbandry. As a result, most researchers have focused on improving the potential of using alternative feed resources derived from the black soldier fly larva. In particular, the utilization of oil from black fly larvae is a byproduct of the industry. The aim of this study was to investigate the influence of black soldier fly larva oils and the proportion of roughage-to-concentrate ratios on gas kinetics, rumen characteristics, degradability, and mitigate CH_4_ production by using in vitro gas production techniques. The in vitro investigation used a completely randomized design (CRD) with a 2 × 4 factorial arrangement. The level of R:C ratios (60:40 and 40:60) were factor A, while BSFO levels (0, 2, 4, and 6% of DM) were factor B. Under this investigation, the combined impact of R:C ratio and BSFO on the kinetics of gas and accumulative gas production was found to be significant (*p* < 0.01). After 4 h of incubation, the pH and ammonia-nitrogen (NH_3_-N) concentration were found to be impacted by the inclusion of BSFO levels at different R:C-ratios (*p* < 0.01). Moreover, after 4 and 8 h of incubation, supplementing the BSFO at 4% with the level of R:C ratio at 40:60 resulted in a significant reduction in the amount of CH_4_ in the rumen (*p* < 0.05). However, the inclusion of BSFO levels at different R:C ratios had no effect on the degradability of DM after 12 and 24 h of incubation (*p* > 0.05), whereas increasing the concentration of BSFO in concentrate at 6% reduced the DM degradability after 24 h of incubation (*p* < 0.05). Furthermore, adding BSFO to the diet at various R:C ratios enhanced the propionate (C3) concentration, with the highest level observed with the level of R:C ratio at 40:60 and 4% BSFO inclusion (*p* < 0.05). To summarize, the addition of BSFO at 4% with a 40:60 of R:C ratio increased C3 levels, decreased CH_4_ emission, and preserved DM degradability. A R:C ratio of 40:60 could improve the total volatile fatty acids and digestibility. Moreover, the inclusion of 6% BSFO at different R:C ratios lowered the in vitro dry matter digestibility, in vitro organic matter digestibility, NH_3_-N, and protozoal populations.

## 1. Introduction

A greenhouse gas (GHG) released by livestock is methane (CH_4_), which accounts for 19% of all emissions [1], 45% of which are related to intestinal fermentation [2]. Methanogenic archaea use hydrogen present in the rumen to reduce CO_2_ to CH_4_ through methanogenesis; this causes the gross energy (GE) absorbed in the meal to decrease by 2 to 12% [3]. The rumen’s anaerobic breakdown of digested organic compound (OM) is the primary pathway for producing CH_4_ as the end product. Thus, the goals of CH_4_ mitigation measures in ruminants have been to gain economic and anti-global warming benefits [4].

Several methods have been attempted to reduce emitting CH_4_ in animals, such as defaunation, the use of chemical inhibitors, and the use of ionophores, which can either indirectly or directly suppress methanogenesis in the rumen; however, no consistently effective approaches have been identified for practical use. One of the alternative interesting topics is the addition of lipids in the diets of cattle. Typically, lipid supplementation is utilized to boost the ration’s energy density [5]. There is significant proof that enteric CH_4_ production is reduced by additional oil-rich meals [6]. By lowering fermentation and CH_4_ production, switching ruminant diets from carbs to lipids has been demonstrated to have an effect on gas generation in the rumen [7,8]. Studies have shown that adding 2 to 6% of lipids to the diets of ruminants can lead to reductions in CH_4_ emissions ranging from 7.2% to 21.4% [9,10]. Numerous causes, including the substitution of rumen fermentable organic compounds in feed, a decline in ruminal protozoa and methanogen, and the biohydrogenation of unsaturated fatty acids, are credited with this decrease [11].

Oil is a substance that is primarily abundant in insects. For ruminants, insects have lately been proposed as a sufficient, sustainable, and novel source of protein and fat [12], with no detrimental effects on rumen characteristics or animal performance [13]. It has been discovered that the concentration of ether extract in black soldier fly larvae oils (BSFO) ranges from 15.0 to 34.8% dry matter [14]. Several writers have investigated and reported on the fatty acid composition of oils derived from BSFO [15,16]. To our knowledge, there is currently no research available on the influence of insect oils on rumen properties, degradability, and the reduction of CH_4_ production.

The purpose of the present study was to investigate the effect of black soldier fly larvae oils and the roughage-to-concentrate ratio on gas patterns, rumen characteristics, degradability, and CH_4_ reduction utilizing in vitro gas production techniques.

## 2. Materials and Methods

### 2.1. Black Soldier Fly Larvae Oil Preparation and Design

To maintain the nutritional and chemical quality, the clean and dried black soldier fly larvae (BSF) were subjected to press-defatting at 45 °C to 48 °C using an NF-80 cold press (Karaerler, Ankara, Turkey), and the process was carried out by BSFLY Company Ltd., Udonthani, Thailand.

The current experiment was conducted using a gas generation method over a range of incubation times. Using a completely randomized design with three replication runs (CRD), a 2 × 4 factorial experiment was conducted. The experimental diets consisted of two different roughage-to-concentrate (R:C) ratios (60:40 and 40:60) and four different levels of BSFO concentration (0, 2, 4, and 6% DM). As a roughage source, rice straw was used. Table 1 lists the components and nutrients found in both the concentrates and rice straw.

### 2.2. Animals and Rumen Fluid Inoculum

For rumen fluid supply, Thai native steers (2.0–2.3 years old) weighing 350 ± 10 kg were employed. For 14 days, at 7:30 and 15:30, the cattle were fed with concentrate mixture (14.0% crude protein (CP) and 75% total digestible nutrient) at 1% of their weight and rice straw was fed on an ad libitum basis. Each animal was housed in a separate corral and had unrestricted access to fresh, clean water as well as mineral blocks. Before morning feeding, rumen fluids from each cow were also collected using a pump connected to a stomach tube. The fluids were transferred to thermos flasks that had been preheated and had an O_2_-free headspace before being transported anaerobically to the lab at 39 °C and used as inoculum after passing through four layers of cheesecloth.

### 2.3. Fermentation Substrates In Vitro

Artificial saliva was prepared on the day of conduction, prior to the collection of rumen fluids. A two-set inoculum mixture in thermos flasks containing artificial saliva and rumen fluid at a ratio of 2:1 was performed. The rice straw and concentration samples, weighing 0.5 g each, were placed into 50 mL serum vials. Four replications with five blanks were created for each treatment. For the in vitro gas generation test, the bottles were closed with rubber disclosures and crimp caps, which were then incubated for 96 h at 39 °C. Every three hours, the bottles were gently shaken during the incubation phase. Three 50 mL glass bottles, one in quadruplicate and one in blank, each holding 0.5 g of substrate on a dry matter basis, were set up, flushed with carbon dioxide, and preheated to 39 °C. The bottles were divided into three sets: the first set (4 bottles × 8 treatments × 4 bottles of blank) was used for gas kinetics and gas production measurement (incubated for 96 h), the second set (4 bottles × 8 treatments × 2 observation times at 4 and 8 h) for the measurement of ruminal parameters including pH, ruminal ammonia-nitrogen (NH_3_-N), volatile fatty acids, and protozoa, and the third set (4 bottles × 8 treatments × 2 observation times at 12 and 24 h × 4 bottles of blank) was used for the determination of nutrient digestibility.

### 2.4. Measurements and Chemical Analysis

The samples of rice straw and concentrate were put through a 1-mm screen on a Cyclotech Mill from Tecator in Hoganas, Sweden, dried for 72 h at 60 °C, then analyzed in vitro for chemical composition and gas generation. According to the AOAC International Method [17], the following quantities were measured and conveyed with any leftover ash included: dry matter (DM), ether extract (EE), organic matter (OM), and acid detergent fiber (ADF). The α-amylase and sodium sulfite and were used to compute the neutral detergent fiber (NDF) in accordance with the methodology of Van Soest et al. [18] (Sigma no. A3306, Sigma Chemical Co., St. Louis, MO, USA). According to the AOAC [17], the amount of CP in the feed samples was measured using a Leco combustion nitrogen analyzer (Leco CN628 Carbon/Nitrogen Analyzer, Leco Instruments Inc., St. Joseph, MI, USA). The fatty acid content of BSFO was assessed using gas chromatography with a flame ionization detector (GC-FID) (Thermo Scientific Trace GC Ultra) and a WCOT fused silica column (100 m, 0.25 mm i.d., 0.2 m f.t., Coating Select Fame, Varian, Houten, The Netherlands). Table 1 shows the results as a percentage of total fatty acids. A pressure transducer and calibrated syringe were used at many moments in time (0.5, 1, 2, 4, 6, 8, 12, 18, 24, 36, 48, 72, and 96 h) to assess gas generation right after incubation. After inserting a fitted syringe with needle No. 24 into the rubber stopper of fermentation bottles, the bottles were placed in an incubator. The pressure transducer’s metering system was made up of a 3-way stopcock valve, a mechanical pressure gauge, a glass syringe, and a needle. The pressure within the serum bottles was monitored through the transducer linked to the 3-way valve, while the amount of gas produced was measured with the glass syringe. The third port of the valve was connected to a hose with a needle for puncturing the butyl rubber stopper plug on the injection bottle.

The pH levels of 64 bottles of fermented inoculum were determined at 4 and 8 h after inoculation using 32 samples taken at each time. The liquid was then filtered through Grade 40 Cheesecloth. The frozen rumen fluid was thawed and centrifuged at 16,000 g for 10 min to determine NH_3_-N and the volatile fatty acid contents (VFAs). The VFA proportion was determined using gas chromatography, as outlined in the approach by So et al. [19] using an altered technique of Osaki et al. [20]. According to the Fawcett and Scott method [21], 40 microliters of centrifuged rumen liquid, 2500 microliters of phenol color reagent, and 2000 microliters of alkaline hypochloride reagent were combined in a 15 mL test tube using a vortex mixer. A blue reaction was produced when the mixture was vortexed and then incubated for 10 min at 37 °C in a shaking water bath. A second 630 nm UV/Vis spectrophotometric analysis of the mixture followed. To assess the rumen’s microorganisms, a 1 mL sample of the serum was mixed thoroughly with 9 mL of formaldehyde after being incubated for 4 and 8 h. Ruminal protozoa was quantified using a hemocytometer (Boeco, Hamburg, Germany) and a manual counting technique [22]. A leak-proof syringe was used to extract 10 mL of gas from the fermented bottle’s headspace, and gas chromatography was used to determine the amount of CH_4_ in the sample at 4 and 8 h after incubation (Shimadzu Corporation, Kyoto, Japan).

After filtering the contents through a pre-weighed glass filter crucible of 50 mL, the material was incubated for 12 and 24 h (4 samples per treatment) before determining its in vitro dry matter digestibility (IVDMD) [23]. The NDF concentration of the indigestible residues in the test bottles was determined in order to compute the in vitro true digestibility (IVTD) [18] using the following equations.

IVTD = 100 − [(100 − NDFD) × (NDF/100)],

where NDF = neutral detergent fiber (% of DM), IVTD = in vitro true digestibility (% of DM), and NDFD = neutral detergent fiber digestibility (% of NDF).

To determine the amount of organic matter (OM) and the percentage of OM digestibility (IVOMD), the glass filter crucible was heated at 550 °C for 6 h. The residue data were then used to make these determinations.

### 2.5. Statistical Analysis

The cumulative gas production curve was calculated using the Schofield [24] model.

Gas production = b × [1 − exp^−c(t − L)^],

where b is the final asymptotic gas volume corresponding to fully digested substrate (mL/g DM), t is the incubation time (h), c is a rate constant (units time 1), and L is a discontinuous lag term (h).

The PROC GLM of SAS [25] was used to evaluate the in vitro study’s data in accordance with a 2 × 4 factorial in a completely randomized system. The model is:

Y_ijk_ = µ + a_i_ + b_j_ + ab_ij_ + ε_ijk,_

where Y_ijk_ are the response variances; µ is the overall mean, ai are the R:C ratio levels at 60:40 and 40:60 (i, 1–2), b_j_ are the BSFO levels at 0, 2, 4, and 6% of DM (j, 1–4), ab_ij_ are the interaction effects, and ε_ijk_ are the residues. The means of the response variances were also presented, along with the standard error of the mean. Mean values of each individual run were used as the experimental unit. With a significance level of *p* < 0.05, the least significant difference (lsd) was utilized to examine statistical differences between treatment means.

## 3. Result

### 3.1. Nutritional Composition of Diet

Table 1 lists the ingredients and chemical composition of concentrate diet and rice straw. The protein level of the concentrate diets, which ranged from 15.05 to 15.45% DM in each group, was almost the same, and urea was given to modify the CP content, and rice straw was used as a source of roughage in a substrate that contained CP at a concentration of 2.54% DM. The EE contents of the inclusion of 0, 2, 4, and 6% BSFO in the experimental diets were 2.05, 4.02, 6.03, and 7.96, respectively, which progressively increase when BSFO is added from 0 to 6%. Additionally, adding 0, 2, 4, and 6% BSFO to the feed increased the amount of GE, from 16.98 to 17.67, favoring gross energy density.

### 3.2. Kinetics of Gas Production

**Figure 1:**
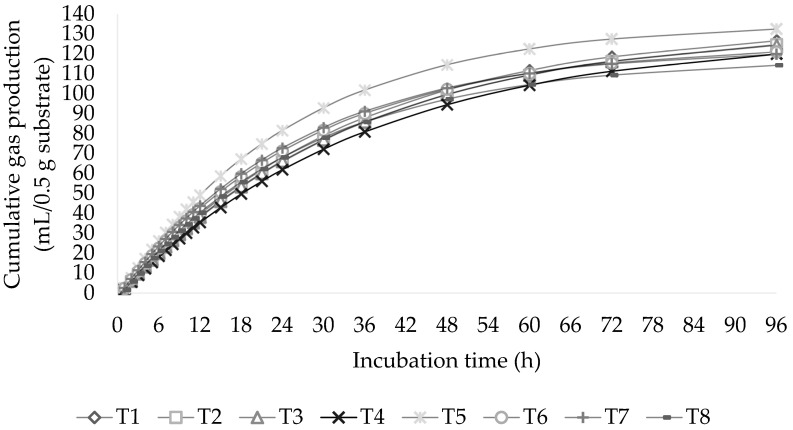
Effect of roughage-to-concentrate (R:C) ratio level combined with black soldier fly oil (BSFO) level on cumulative gas during incubation times.

The calculated parameters for the tested substrates based on the kinetics of producing gases models are shown in Table 2. At 96 h of incubation, a relationship among the R:C ratio and increasing the BSFO for asymptotic gas production (b) and gas accumulation was observed (Figure 1; *p* < 0.01). According to the findings, the asymptotic gas production and overall gas production were at their peak in the R:C ratio (40:60) with BSFO included at 0%. The rate of gas generation (c) and the discrete lag time (L) before gas production did not interact at the R:C ratio and BSFO levels (*p* > 0.05). However, the discrete lag time was reduced (*p* < 0.01) when a significant amount of concentrate diet was administered.

### 3.3. In Vitro Ruminal Fermentation and CH_4_ Concentration

Table 3 illustrates the influence of R:C ratio and BSFO level on NH_3_-N concentration, pH, CH_4_ output, and protozoal population after 4 and 8 h of incubation using an in vitro gas technique assay. After 4 h of incubation, there was a significant difference detected (*p* < 0.01) among the inclusion of BSFO levels at different R:C-ratios for pH and NH_3_-N concentration, and the values ranged from 6.95 to 7.03 and 18.42 to 22.41 mg/dL, respectively. The inclusion of BSFO levels at different R:C ratios did not have an effect on NH_3_-N and pH during 8 h of incubation (*p* > 0.05). However, the level of R:C ratio at 40:60 dramatically reduced pH, whereas increasing the amount of BSFO in the diet up to 2% significantly decreased the impact (*p* < 0.01) on ruminal NH_3_-N, with the lowest values of 17.28 mg/dL for the inclusion of BSFO at 6%. The concentration of CH_4_ was significantly reduced by enhancing doses of BSFO in various roughage-to-concentrate ratios. The amount of CH_4_ was at its lowest (2.45 mL/1 g) at 4 h after incubation when the level of R:C ratio at 60:40 in 4% BSFO inclusion (*p* < 0.01), and at its lowest (6.30 mL/1 g) at 8 h after incubation when the level of R:C ratio was at 60:40 in 4% BSFO inclusion (*p* < 0.05). For protozoal populations at 4 and 8 h after incubation, there was no relationship between the level of R:C ratio and BSFO inclusion (*p* > 0.05). Nonetheless, adding up to 2% BSFO considerably reduced the impact (*p* < 0.01) on protozoal numbers at 4 and 8 h, with the lowest values being 2.50 × 10^6^ and 3.00 × 10^6^ cells/mL, respectively.

### 3.4. In Vitro Degradability

Table 4 lists the impact of the roughage-to-concentrate ratio and the BSFO level on the in vitro digestibility of DM, OM, and IVTD. The in vitro digestibility was unaffected by the level of R:C ratio and BSFO inclusion (*p* > 0.05), except that IVOMD at 12 h and IVTD at 24 h after incubation have the lowest values at 36.42% and 76.51% DM, respectively, when the level of R:C ratio was 60:40 in BSFO inclusion at 6% (*p* < 0.05). The IVDMD, IVOMD, and IVTD at various hours of incubation times were increased when a high quantity of concentrate diet was provided (*p* < 0.05). In addition, the presence of BSFO at 6% decreased the values of the in vitro digestibility of DM and OM at 24 h, which were 39.53 and 46.70% DM, respectively (*p* < 0.01).

### 3.5. Ruminal Volatile Fatty Acid Concentration

Table 5 illustrates the influence of the R:C ratio and BSFO on total VFAs, acetate (C2), propionate (C3), and butyrate (C4) concentrations. No changes (*p* > 0.05) existed between the inclusion of BSFO levels at different R:C ratios on total VFAs and profiles at 4 h. Nevertheless, increasing the amount of concentrate diet from 40% to 60% enhanced total VFAs by 61.08 mmol/L (*p* < 0.01). After 8 h of incubation, the level of R:C ratio at 40:60 caused a decrease of C2 in rumen fluids of approximately 2.28% when compared with the level of R:C ratio at 60:40, whereas increasing the amount of BSFO in the diet up to 2% significantly decreased the effect (*p* < 0.01) on the concentration of C2 with the highest values of 68.33 and 68.85% for inclusion of BSFO at 4 and 6%, respectively. At 8 h, there were relationships (*p* < 0.05) among the level of R:C ratio and BSFO on C3 and C4. The level of R:C ratio at 40:60 in BSFO inclusion at 4 and 6% shows the highest C3 at approximately 21.10 and 20.67%, respectively. In addition, the C4 concentration was lowest (11.47%) when the level of R:C ratio at 40:60 with 2% BSFO inclusion (*p* < 0.05).

## 4. Discussion

### 4.1. Production of Gas and Kinetics

There is evidence that the inclusion of BSFO in a ruminants diet affects the kinetics of gas in the rumen [26]. The addition of oil tends to decrease the gas generation rate and increase the duration of the lag time, or the time it takes for gas to start being produced after feeding. In this study, higher amounts of BSFO in the various R:C ratio levels reduced asymptotic gas production and total gas. This might be a result of the concentrate diet’s high oil content, which slows the rate of feed fermentation in the rumen and reduces gas output. Oil can also affect the physical properties of the rumen, such as the viscosity and flow rate, which can influence the rate and extent of digestion [27]. The combination of garlic powder and coconut oil in in vitro studies, according to Kongmun et al. [28], results in gas generation. In comparison to the control group, adding coconut oil at various doses reduced gas accumulation. Similarly, Kang et al. [29] discovered that using krabok seed oil as a rumen stimulant in a gas production system significantly reduced gas values and kinetics.

### 4.2. In Vitro Ruminal Fermentation and CH_4_ Concentration

Ruminal pH is an important metric because it indicates the internal balance of the rumen ecosystem. Ruminants typically maintain well-balanced conditions in order to maintain a ruminal pH ranging from 6.5 to 7 [30]. The current investigation found that, when concentrate intake increased, the ruminal pH dropped. The rumen’s pH was found to be at its lowest when the level of R:C ratio was 40:60 and BSFO was excluded. The observed decrease in pH could be attributed to an elevated concentration of fermentable carbohydrates in the concentrate diet, which undergo rapid fermentation. According to Ramos et al. [31], consuming feed high in concentrated substances caused the ruminal pH to significantly drop, the rate of metabolism to slow down, and the activity of cellulolytic bacteria to decrease. Subacute ruminal acidosis (SARA) and the control of bacterial growth are both influenced by the daily variation of ruminal pH. The level of pH in the rumen shows that stable pH is necessary for healthy rumen ecology, metabolism, and microbial development.

Microbial protein synthesis is mostly nitrogen-dependent, and ammonia can be the primary supply of nitrogen for bacterial development. At 4 h after incubation, the NH_3_-N content in the present study varied between 18.42 and 22.41 mg/dL. The maximum NH_3_-N concentration was reported when the diet contained 0% to 4% BSFO in the level of R:C ratio at 40:60. When compared to a low-concentrate diet, a high-concentration feed ratio with a higher CP may result in higher amounts of NH_3_-N due to increased microbial breakdown. Maintaining an NH_3_-N concentration in the 15–30 mg/dL range can improve rumen ecology in areas such as feed intake, bacterial protein production, and digestibility [32]. In contrast, a lack of NH_3_-N can slow bacterial development. The high content of ruminal NH_3_-N in the high-concentrate diet is explained by a dynamic balance among NH_3_-N production and consumption by rumen bacteria [33,34]. The observation of large amounts of NH_3_-N in the stomach of ruminants indicates that the ruminants diet contains an adequate amount of accessible nitrogen, which is most likely due to the diet’s high concentrate concentration. According to Musco et al. [33], the higher concentration of ruminal NH_3_-N in the high-concentrate diet could also be due to the higher presence of rumen-degradable protein. In addition, the findings imply that increasing the amount of oil in the diet may reduce NH_3_-N concentrations in the rumen, probably due to a decrease in protozoa populations. This finding is consistent with a prior study that found that adding plant oil reduced rumen ammonia concentrations. Ammonia is typically formed by the degradation of bacterial proteins, which is frequently related to the presence of protozoa. Several studies show that using essential oils can reduce ammonia concentrations in in vitro systems. According to Castillejos et al. [35], essential oils can interact with the bacterial cell membrane, preventing the growth of specific strains and ultimately resulting in a drop in ammonia concentration [36].

The amount and type of dietary lipids, as well as the mix of the diet, all influence the changes that take place in the rumen ecology [37]. The amount and type of dietary lipids, as well as the food mix, all influence the changes that occur in the rumen ecology, which explains why the lipid’s influence on the quantity and/or function of the microorganisms varies [11]. The current study’s protozoa population changed as a result of the inclusion of feeding BSFO. This is due to BSFO’s high lauric acid concentration, which has significant antiprotozoal characteristics and, as a result, has a variety of impacts on the development of fermentation end products and CH_4_ reduction in the rumen. Due to the impact of medium-chain fatty acids (MCFAs), prior study has revealed that a large amount of vegetable oil supplementation could decrease the rumen protozoal population in ruminants [11]. In addition, lauric (C12) and myristic (C14) acids in olive oil have a detrimental effect on protozoal membranes, which can reduce their fibrolytic activity, similar to what was found in Matsumoto et al. [38].

Reduced protozoa numbers are frequently accompanied by a decrease in CH_4_ generation as a result of increased dietary fat concentrations. In this experiment, increasing the BSFO inclusion reduced CH_4_ output. It has been demonstrated that adding oil or fat from a variety of sources can reduce the release of CH_4_ from cattle [11]. By decreasing the number or activity of methanogens, partially eliminating protozoa (which serve as the host for some methanogens), and reducing nutrient breakdown and the process of fermentation (which both promote hydrogen creation and, in the case of fatty acids that are unsaturated, act as substitute hydrogen sinks), oil can be used to inhibit methanogenesis [39]. The results of the study show that medium-chain fatty acids, long-chain fatty acids, saturated fatty acids, and unsaturated fatty acids are all effective at reducing the gastrocolic emissions of CH_4_ [40].

### 4.3. In Vitro Degradability

The digestibility of DM, OM, and true digestibility (TD) were enhanced as the percentage of concentrate was increased. This may be due to the fact that a concentrate diet might provide additional nutrients for the growth of rumen bacteria, thereby improving rumen fermentation. The improvement in DMD, OMD, and TD caused by a higher proportion of concentrate in the diet is a clear indication of concentrate components’ improved digestibility compared to treated straw [41]. These findings are comparable with those of Chen et al. [42], who found that cows fed a high concentrate diet had a considerable increase in nutritional digestibility. In contrast, Calabro et al. [43] compared several feeds and found higher OM in vitro digestibility as oil content increased. Moreover, oil supplementation in ruminant diets has been shown to have both positive and negative effects on rumen fermentation and digestion. In the current experiment, the digestibility of DM decreased with increasing BSFO levels. The reduction in the digestibility of fiber promoted by vegetable oil is attributed to two main effects: the physical effect of coating the feed particles and the toxic effect on the rumen microorganisms. These findings are consistent with those of Arcos-lvarez et al. [44], who found a decrease in digestible NDF intake at the two highest levels of olive oil inclusion (4 and 6%). Van-Cleef et al. [45] discovered that a 6% inclusion level of vegetable oils reduced the digestion of DM and fiber. Similarly, Sutton et al. [46] showed a 76% reduction in digestion in the forestomach and a 30% reduction in the overall tract when 40 g coconut oil was supplemented per sheep per day. However, in the present work, it seems that the slightly low in vitro DM and OM digestibilities might be attributed to the diet’s slightly higher fiber content, which may have limited digestibility [47]. Concentrate diets including NDF and ADF range from 19.9 to 22.48% and 12.37 to 14.33% DM, respectively, whereas rice straw contained 72% NDF and 50% ADF. Furthermore, fat addition may contribute to somewhat worse feed digestion.

### 4.4. Ruminal Volatile Fatty Acid Concentration

Increasing the quantity of concentrate in the diet from 40% to 60% resulted in a 13.21% increase in total volatile fatty acids. This could be owing to the presence of highly degradable carbohydrates, particularly starch, in the concentrate, which is consistent with the findings of Cherdthong et al. [48]. Furthermore, Phesatcha et al. [41] found that raising the concentrate ratio in the diet to 60% enhanced VFAs and C3, while decreasing C2 and the acetate to propionate (C2:C3) ratio.

Additionally, the propionate molar proportion constantly increased as BSFO increased, increasing the ratio of a concentrate diet to 60%. According to a previous study, adding fish oil raises the molar proportions of C3, C4, and isobutyric acids [11,49]. While our findings are largely compatible with these studies, the maximum concentration of C3 was reported with a 6% level of BSFO, which could be attributed to BSFO lipolysis-induced glycerol release in the rumen. Glycerol is quickly fermented and transformed into C3, accounting for 35% to 69% of its production [36,44]. Also, the C2:C3 ratio decreases as glycerol is converted to C3. The lipolysis process may also release unsaturated fatty acids, sometimes in excess of their biohydrogenation capacity, without affecting the fermentability of the rumen [37,45]. This causes a rise in the production of C3, which competes with metabolic hydrogen, a byproduct of fermentation, in the body. Similar to this, Mapato et al. [50] showed that supplementing with 6% sunflower oil decreased the ratio of C2:C3 while increasing the molar proportion of ruminal acetate and increasing the molar proportion of C3.

## 5. Conclusions and Recommendation

In conclusion, supplementation of BSFO at 4% at different R:C ratios enhanced C3 concentration and reduced CH_4_ production. An R:C ratio of 40:60 may be beneficial for digestibility and total volatile fatty acids; however, the R:C ratio of 60:40 was ineffective. In addition, the inclusion of 6% BSFO at different R:C ratios lowered the DMD, OMD, NH_3_-N, and protozoal populations. However, to examine how the R:C ratio and BSFO impact actual feeding regimens, and in vivo study is required.

## Figures and Tables

**Table 1:** Ingredient and chemical composition of concentrate diet and rice straw (%DM).

Item	BSFO 0%	BSFO 2%	BSFO 4%	BSFO 6%	Rice Straw
Ingredients, % DM					
Cassava chips	41.0	40.0	40.0	39.0	
Corn meal	15.0	14.0	14.0	14.0	
Rice bran	10.0	10.0	9.0	9.0	
Soybean meal (SBM)	7.0	7.0	7.0	7.0	
Dried brewers’ grains	11.0	11.0	10.5	10.0	
Palm kernel meal	11.0	11.0	10.4	9.8	
Black soldier fly oil (BSFO) ^1^	0.0	2.0	4.0	6.0	
Vitamin and mineral premix ^2^	1.0	1.0	1.0	1.0	
Urea	1.0	1.0	1.1	1.2	
Sodium chloride	1.0	1.0	1.0	1.0	
Molasses	2.0	2.0	2.0	2.0	
	Chemical composition				
Dry matter, %	88.39	88.34	88.59	88.11	88.57
Organic matter, % DM	94.57	94.77	95.05	95.14	89.56
Crude protein, % DM	15.21	15.45	15.05	15.05	2.54
Ether extract, % DM	2.05	4.02	6.03	7.96	0.56
Neutral detergent fiber, % DM	21.23	22.48	21.61	19.90	72.89
Acid detergent fiber, % DM	13.36	14.33	13.33	12.37	50.14
Gross energy (MJ)	16.98	17.44	17.51	17.67	14.67

^1^ Black soldier fly larvae oil contains the following fatty acid composition: 40.1% Lauric acid (C12: 0), 16.3% Oleic acid (C18:1 *n*-9), 15.0% Linoleic acid (C18:2 *n*-6), 13.2% Myristic acid (C14:0), and 13.7% Palmitic acid (C16:0) of the total fatty acids, respectively. ^2^ Contains per kilogram premix: 10,000,000 IU vitamin A; 70,000 IU vitamin E; 1,600,000 IU vitamin D; 50 g iron; 40 g zinc; 40 g manganese; 0.1 g cobalt; 10 g copper; 0.1 g selenium; 0.5 g iodine.

**Table 2:** Effect of roughage-to-concentrate (R:C) ratio level combined with black soldier fly oil (BSFO) level on gas kinetics and cumulative gas at 96 h after incubation.

Treatment	R:C Ratio	BSFO (%)	Gas Kinetics (mL/0.5 g)			Cumulative Gas (mL/g DM Basis)
			b	c	L	
T1	60:40	0	134.47 ^a^	0.03	0.333	126.57 ^b^
T2	60:40	2	132.83 ^ab^	0.03	0.467	124.60 ^bc^
T3	60:40	4	134.60 ^a^	0.03	0.367	126.12 ^b^
T4	60:40	6	129.17 ^bc^	0.03	0.367	120.00 ^c^
T5	40:60	0	135.33 ^a^	0.13	0.533	132.11 ^a^
T6	40:60	2	128.37 ^c^	0.04	0.533	124.56 ^bc^
T7	40:60	4	123.13 ^d^	0.04	0.433	119.83 ^c^
T8	40:60	6	117.75 ^e^	0.04	0.650	114.26 ^d^
SEM			1.359	0.032	0.072	1.510
Main effects						
R:C Ratio	60:40		132.77 ^a^	0.03	0.54 ^b^	124.32
	40:60		126.15 ^b^	0.06	0.38 ^a^	122.69
BSFO (%)	0		134.90 ^a^	0.08	0.51	129.34 ^a^
	2		130.60 ^b^	0.03	0.50	124.58 ^b^
	4		128.87 ^b^	0.03	0.43	122.97 ^b^
	6		123.46 ^c^	0.03	0.40	117.13 ^c^
Significance of main effect and interaction						
R:C Ratio			<0.01	0.1826	<0.01	0.1457
BSFO (%)			<0.01	0.3875	0.3982	<0.01
Interaction			<0.01	0.4100	0.3842	<0.01

^a–e^ Means in the same column with different lowercase letters differ (*p* < 0.05); b = the final asymptotic gas volume corresponding to fully digested substrate (mL/g DM); c = a rate constant (units time 1); L = a discontinuous lag term (h).

**Table 3:** Effect of roughage-to-concentrate (R:C) ratio level combined with black soldier fly oil (BSFO) level on ruminal pH, ammonia-nitrogen (NH_3_-N) concentration, protozoal population, and methane production.

Treatment	R:C Ratio	BSFO (%)	pH		NH_3_-N (mg/dL)		Methane (mL/1 g Dry Matter Substrate)		Protozoa (×10^6^ Cell/mL)	
			4 h	8 h	4 h	8 h	4 h	8 h	4 h	8 h
T1	60:40	0	7.03 ^a^	6.94	19.84 ^b^	20.40	4.50 ^a^	9.45 ^b^	8.00	8.00
T2	60:40	2	6.97 ^bc^	6.94	19.14 ^b^	20.25	4.60 ^a^	8.65 ^bc^	4.00	4.00
T3	60:40	4	6.97 ^bc^	6.92	18.42 ^b^	17.53	2.45 ^c^	9.00 ^c^	3.00	6.00
T4	60:40	6	6.98 ^bc^	6.96	18.96 ^b^	17.01	3.30 ^b^	7.30 ^bcd^	3.00	4.00
T5	40:60	0	6.95 ^c^	6.89	22.41 ^a^	20.67	4.55 ^a^	11.70 ^a^	9.00	10.00
T6	40:60	2	6.97 ^bc^	6.87	21.95 ^a^	18.18	4.55 ^a^	7.30 ^bcd^	5.00	6.00
T7	40:60	4	7.00 ^abc^	6.88	21.52 ^a^	17.22	3.60 ^b^	6.30 ^d^	2.00	4.00
T8	40:60	6	7.02 ^abc^	6.87	18.96 ^b^	17.01	2.65 ^c^	6.40 ^d^	3.00	2.00
SEM			0.016	0.015	0.389	0.582	0.279	0.659	1.061	1.173
Main effects										
R:C Ratio	60:40		6.99	6.94 ^a^	19.19 ^b^	18.80	3.71	8.60	4.50	5.50
	40:60		6.98	6.88 ^b^	21.19 ^a^	18.20	3.84	7.93	4.75	5.50
BSFO (%)	0		6.99	6.92	21.13 ^a^	20.51 ^a^	4.53 ^a^	10.58 ^a^	8.50 ^a^	9.00 ^a^
	2		6.97	6.91	20.54 ^a^	19.73 ^a^	4.58 ^a^	7.98 ^b^	4.50 ^b^	5.00 ^b^
	4		6.98	6.90	20.28 ^a^	17.38 ^b^	3.03 ^b^	7.65 ^b^	2.50 ^b^	5.00 ^b^
	6		7.00	6.92	18.91 ^b^	17.28 ^b^	2.98 ^b^	6.85 ^b^	3.00 ^b^	3.00 ^b^
Significance of main effect and interaction										
R:C Ratio			0.6650	<0.01	<0.01	0.2025	0.3555	0.1855	0.7475	1.00
BSFO (%)			0.2900	0.7099	<0.01	<0.01	<0.01	<0.01	<0.01	<0.01
Interaction			<0.01	0.4157	<0.01	0.591	<0.01	<0.05	0.7520	0.2018

^a–d^ Means in the same column with different lowercase letters differ (*p* < 0.05).

**Table 4:** Effect of roughage-to-concentrate (R:C) ratio level combined with black soldier fly oil (BSFO) level on the in vitro degradability of nutrients.

Treatment	R:C Ratio	BSFO (%)	IVDMD (% DM)		IVOMD (% DM)		IVTD (% DM)	
			12 h	24 h	12 h	24 h	12 h	24 h
T1	60:40	0	31.08	37.34	39.90 ^bc^	45.69	73.39	79.59 ^b^
T2	60:40	2	29.63	36.39	38.29 ^dc^	44.40	73.45	79.48 ^b^
T3	60:40	4	28.59	37.09	37.26 ^d^	45.19	73.55	78.70 ^b^
T4	60:40	6	27.90	35.61	36.42 ^d^	43.52	74.14	76.51 ^c^
T5	40:60	0	35.00	45.74	41.93 ^ab^	52.27	83.72	84.75 ^a^
T6	40:60	2	34.10	45.24	40.85 ^ab^	51.79	83.81	84.16 ^a^
T7	40:60	4	35.14	45.49	42.12 ^ab^	51.80	84.12	84.26 ^a^
T8	40:60	6	35.74	43.46	43.00 ^a^	49.89	83.98	85.45 ^a^
SEM			0.684	0.264	0.704	0.286	0.606	0.482
Main effects								
R:C Ratio	60:40		29.30 ^b^	36.61 ^b^	37.97 ^b^	44.70 ^b^	73.63 ^b^	78.57 ^b^
	40:60		35.12 ^a^	44.98 ^a^	41.97 ^a^	51.44 ^a^	83.93 ^a^	84.66 ^a^
BSFO (%)	0		33.04	41.54 ^a^	40.92	48.98 ^a^	77.53	82.17
	2		31.12	40.82 ^b^	39.57	48.09 ^b^	78.63	81.82
	4		31.87	41.29 ^ab^	39.69	48.49 ^ab^	78.83	81.48
	6		31.82	39.53 ^c^	39.71	46.70 ^c^	79.06	80.98
Significance of main effect and interaction								
R:C Ratio			<0.01	<0.01	<0.01	<0.01	<0.01	<0.01
BSFO (%)			0.2621	<0.01	0.2605	<0.01	0.1279	0.1256
Interaction			0.0604	0.3689	<0.05	0.3674	1.00	<0.01

^a–d^ Means in the same column with different lowercase letters differ (*p* < 0.05); IVDMD = in vitro dry matter digestibility; IVOMD = in vitro organic matter digestibility; IVTD = in vitro true digestibility.

**Table 5:** Effect of roughage-to-concentrate (R:C) ratio level combined with black soldier fly oil (BSFO) level on volatile fatty acid (VFA) concentrations.

Treatment	R:C Ratio	BSFO (%)	Total VFAs (mmol/L)		Acetate (%)		Propionate (%)		Butyrate (%)	
			4 h	8 h	4 h	8 h	4 h	8 h	4 h	8 h
T1	60:40	0	42.55	52.85	72.63	72.53	14.92	15.54 ^d^	12.45	11.93 ^ab^
T2	60:40	2	41.03	53.54	72.54	70.35	14.78	16.95 ^c^	12.68	12.70 ^a^
T3	60:40	4	42.77	51.69	72.76	69.30	14.86	17.95 ^bc^	12.68	12.76 ^a^
T4	60:40	6	41.80	53.97	72.31	70.07	15.12	18.38 ^b^	12.59	11.55 ^b^
T5	40:60	0	40.17	58.76	71.36	70.57	15.15	16.97 ^c^	13.22	12.46 ^ab^
T6	40:60	2	41.76	61.53	72.54	70.39	15.06	18.14 ^b^	12.67	11.47 ^b^
T7	40:60	4	41.99	62.49	72.04	67.36	15.19	21.10 ^a^	12.88	11.54 ^b^
T8	40:60	6	40.30	61.55	72.26	67.63	15.03	20.67 ^a^	12.83	11.71 ^ab^
SEM			1.235	2.105	0.455	0.547	0.303	0.350	0.234	0.336
Main effects										
R:C ratio	60:40		41.99	53.01 ^b^	72.56	70.56 ^a^	14.92	17.20 ^b^	12.61	12.23
	40:60		41.13	61.08 ^a^	72.05	68.99 ^b^	15.10	19.22 ^a^	12.90	11.79
BSFO (%)	0		41.36	55.80	71.87	71.55 ^a^	15.03	16.26 ^c^	12.91	12.19
	2		41.39	57.54	72.54	70.37 ^b^	14.92	17.55 ^b^	12.68	12.09
	4		42.46	57.09	72.40	68.33 ^c^	14.99	19.52 ^a^	12.80	12.15
	6		41.20	57.76	72.29	68.85 ^c^	15.09	19.52 ^a^	12.74	11.63
Significance of main effect and interaction										
R:C ratio			0.3886	<0.01	0.1459	<0.01	0.4556	<0.01	0.1308	0.0823
BSFO (%)			0.7822	0.7920	0.5599	<0.01	0.9584	<0.01	0.8077	0.3391
Interaction			0.7184	0.7098	0.5835	0.1481	0.9066	<0.05	0.5100	<0.05

^a–d^ Means in the same column with different lowercase letters differ (*p* < 0.05).

## Data Availability

Not applicable.
